# Effects of melatonin implantation on cashmere yield, fibre characteristics, duration of cashmere growth as well as growth and reproductive performance of Inner Mongolian cashmere goats

**DOI:** 10.1186/s40104-015-0023-2

**Published:** 2015-05-27

**Authors:** Chunhui Duan, Jianhai Xu, Changmian Sun, Zhihai Jia, Wei Zhang

**Affiliations:** State Key Laboratory of Animal Nutrition, College of Animal Science and Technology, China Agricultural University, Beijing, 100193 People’s Republic of China; College of Veterinary Medicine, China Agricultural University, Beijing, 100193 People’s Republic of China

**Keywords:** Cashmere goat, Cashmere yield, Fibre characteristics, Growth performance, Melatonin, Reproductive performance

## Abstract

**Background:**

Exogenous melatonin could induce cashmere growth. However, induced growth of cashmere fleece by melatonin implants cannot be combined with the typical growth, resulting in earlier shedding followed by another cycle of cashmere growth. To address this issue, we examine the effects on the cashmere yield, fibre characteristics, and the growth and reproductive performance of cashmere goats of planned administration of melatonin.

**Methods:**

Eighteen half-sib, female goats were assigned to two treatments (*n* = 9) including a control and a treatment where melatonin (2 mg/kg BW) was implanted at the end of April and end of June. Cashmere growth and shedding were observed for approximately 1 year following implantation. Fibre samples were collected monthly to determine cumulative cashmere length. Initiation and cessation dates for cashmere growth as well as the rate of cashmere growth were calculated. Cashmere yield, weight gain of dam, kidding date, litter size, and birth weight were also recorded.

**Results:**

Melatonin implantation increased cashmere yield by 34.5 % (control 553.7 g vs. melatonin 745.0 g; *P* < 0.01), cashmere length by 21.3 % (control 95.2 mm vs. melatonin 115.4 mm; *P* < 0.01), and decreased fibre diameter by 4.4 % (control 14.6 μm vs. melatonin 14.0 μm; *P <* 0.03). In melatonin-treated goats, the average initiation date was earlier than in control goats (May 18, 2013 vs. July 2, 2013; *P* < 0.01) but there was a similar cessation date (March 22, 2014 vs. March 27, 2014). Consequently, the duration of cashmere growth was longer in melatonin-treated goats than in control goats (307 vs.270 days; *P* < 0.01). The final BW, average daily gain, kidding date, litter size, and birth weight were not influenced by melatonin implantation.

**Conclusions:**

These data indicate that melatonin implantation (2 mg/kg BW) on two occasions (late April and June) increased cashmere yield by combining the induced growth of cashmere fleece with the typical growth and decreased the fibre diameter without changing dam growth rate or reproductive performance.

## Introduction

The cashmere obtained from goats (*Capra hircus*), is one of the finest and softest fibres produced by animals and is used exclusively in the production of luxurious textile products [1]. Cashmere, famous for its high performance and rareness, is more expensive than wool with an identical diameter [2] and the demand for it is growing [3]. The majority of cashmere is produced in China, Mongolia, Iran and several other countries, where cashmere production makes an important contribution to the economy [4]. China is the world’s largest producer of cashmere accounting for 50 % of the world’s production of cashmere with about 30 % of this production coming from Inner Mongolian cashmere goats [5]. The Inner Mongolian cashmere goat produces cashmere of the highest quality, including a desirable color (white), brightness, elasticity, and an average fibre diameter under 15 μm [6]. Growth of cashmere in the Inner Mongolian cashmere goat exhibits a seasonal pattern arising from circannual changes in the natural photoperiod with cashmere growth typically starting in July and stopping the following March with shedding of the fleece at the end of April [7].

Research has shown that melatonin is a critical intermediary between photoperiod and cashmere growth [8] and circulating melatonin levels directly affect cashmere growth [9]. It has been confirmed that exogenous melatonin has a positive role on cashmere growth, unfortunately, previous experiments have not been able to show a practical method of increasing cashmere yield. Previous studies have shown that the use of exogenous melatonin could stimulate cashmere growth during the cashmere non-growing period, but also resulted in earlier cashmere shedding followed by another cycle of cashmere growth [9–12]. The consistent conclusion was obtained in Inner Mongolian cashmere goats [13, 14]. We hypothesized that if the period of shedding could be prevented, the extended cashmere growth phase could result in an increase in cashmere production. If it does, it will be of great breakthrough for melatonin regulates cashmere growth. Therefore, the objective of this study was to determine the effects of melatonin implantation during the cashmere non-growing period on cashmere yield, fibre characteristics, and the growth and reproductive performance of Inner Mongolian cashmere goats.

## Material and methods

### Animals and experimental design

This experiment was performed at the YiWei White Cashmere Goat Farm located in the Inner Mongolia Autonomous Region of China (latitude 39°06’N, longitude 107°59’E and at an altitude of 1500 m) from April 30, 2013, to April 30, 2014. All procedures used in this study were approved by the Animal Care and Use Committee of China Agricultural University (Beijing, China).

Eighteen 2.1-year-old lactating, female, half-sib, Inner Mongolian cashmere goats with an initial body weight of 32.92 ± 2.78 kg were combed with a small wooden rake to remove the previous year’s fleece. The goats were then randomly assigned to two groups (*n* = 9) including a control and a treatment where melatonin (2 mg/kg BW; Beijing Kangtai Biological Technology Company, Beijing, China) was implanted on April 30 and June 29. Melatonin was subcutaneously implanted at the base of the ear and the dose applied was based on the work of Yue et al. [15].

During the experimental period, all goats were maintained under natural photoperiodic conditions and the feeding and management was typical of other goats at the cashmere goat farm where goats are kept year round in a desert pasture with occasional supplementary feeding. Goats were provided with grazing and supplementary feeding management from January to June, with 0.275 kg/d concentrate (70 % corn and 30 % condensed feed purchased from Baotou Jiuzhoudadi Biotech Company, Baotou, China) per goat provided in January, 2013 which was gradually increased to 0.4 kg/d in April and subsequently increased to 0.55 kg/d in May and June to meet the needs of the goats during lactation. Goats were grazed on natural pasture without supplementary feeding from July to December. The composition of the primary vegetation in the area was *Caragana stenophylla poiark, Caragana rorsninskii kom*, *Agriopyron cristutum gaertn, Agriopyron cristutum schut, Alium polyrhizum turcz, Artemisia frigidi willd, Artemisia ordosica praschen, Stipa breviflora griseb, Haloxylon ammodendron bunge,* some of which are grazed only by goats [5]. Mating occurred over approximately 30–60 days (October-November, 2013) with parturition during the months of March-April, 2014.

### Data and sample collection

A patch of fleece (30 mm × 30 mm) was shorn at the end of every month from the mid-side of each goat at the skin level. Each clipping was obtained immediately adjacent to the location of the last shearing but was always different from any previously sampled areas. Samples were manually separated into cashmere fibre and guard hair samples. The cashmere fibre samples were stored in sealed polythene bags at room temperature for subsequent analyses of fibre length and diameter.

All goats were individually identified. The body weight of each goat was recorded at the beginning and end of the study. Identification number, kidding date, type of birth, sex, and birth weight were also recorded. Cashmere was harvested at the end of April, 2014 by combing as previously described and was weighed using an electronic scale following harvest.

### Analytical procedures

The cashmere fibre samples were soaked in carbon tetrachloride detergent solution overnight, rinsed thoroughly in deionized water and then dried at 80 °C. The stretched length of the cashmere fibre was measured to the nearest millimeter and the amount of cashmere growth was calculated. The measurements were conducted each month on 100 grab samples randomly selected from the separated cashmere fibres. The diameter of 200 randomly chosen fibre samples was measured using an Optic Fibre Diameter Analyzer (CU-6, Beijing United Vision Technical Company, Beijing, China).

Average cashmere growth rate was determined from the linear portion of cumulative cashmere growth curves, in which a regression line (*Y* = *A* + b*X* where *Y* is the fibre length and *X* is the day of the year) was calculated to develop a model that could explain the cycle of cashmere growth [16]. The slope of the regression line (*b*) describes the average rate of fibre elongation. The initiation date of cashmere growth was determined from the regression equation relating cashmere growth and time as the point of intersection with the *x*-axis (*Y*_min_ = 0). The cessation date was defined as the time when maximum length was achieved, and calculated as the point where *Y*_max_ intersected the regression line relating change in cashmere length and date.

### Statistical analysis

The data were analyzed using the *t*-test procedure of SAS [17]. Statistical differences between initiation and cessation date, rate and phase of cashmere growth were detected by *t*-tests, using estimates of variance described elsewhere [16].

## Results

### Productive performance

The effects of melatonin treatment on goat performance are shown in Table 1. The initial BW, final BW, and average daily gain were not significantly different between the treatments. The yield of cashmere was significantly higher for the melatonin treatment than the control (745.0 vs. 553.7 g; *P* < 0.01). Melatonin implants significantly increased the maximum length (115.4 vs. 95.2 mm; *P* < 0.01) and decreased fibre diameter compared with the control goats (14.0 vs. 14.6 μm; *P* < 0.03).

**Table 1:** Effect of melatonin implantation on productive parameters of Inner Mongolian cashmere goats^1^

Item	Treatment		S.E.M^2^	*P*-value
	Control	Melatonin		
Initial body weight^3^, kg	32.7	33.1	1.53	0.757
Final body weight^3^, kg	37.0	37.9	2.17	0.655
Average daily gain, g	11.9	13.3	3.05	0.648
Cashmere yield, g	553.7^b^	745.0^a^	52.78	<0.01
Maximum length, mm	95.2^b^	115.4^a^	4.55	<0.01
Cashmere fibre diameter, μm	14.6^a^	14.0^b^	0.25	0.026

^1^Values are means of nine replicates per treatment. Means with different superscripts (a and b) within the same row differ (*P* < 0.05)

^2^S.E.M = Standard error of the mean

^3^Initial body weight taken when implants were inserted (i.e. April 30) and final body weight was taken when cashmere production ceased

### Cashmere growth curves and cashmere growth rate

**Fig. 1:**
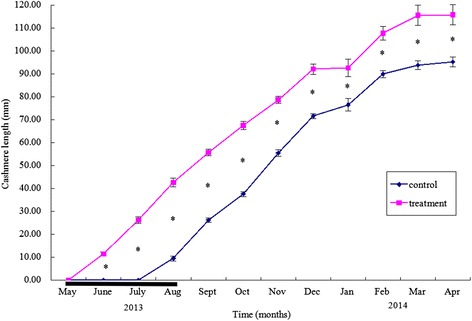
Cumulative cashmere length (mm) for control and melatonin implanted goats (from April to June). Values are means of nine replicates per treatment. Asterisks indicate that differences between treatments were significant (*P* < 0.01). The heavy horizontal line represents the minimum period of continuous release of melatonin

The cashmere growth curves of the control and implanted goats are presented in Fig. 1. There was no change in cashmere length (0 mm) in the control goats from May to July 2013, after which the cashmere began to grow and increased to a maximum by April 2014. In contrast, cashmere length of the treated goats increased steadily from May 2013 until April 2014. The pattern of cashmere growth in treated goats was similar to that of control goats from July 2013 to April 2014, but the cashmere length in treated goats was longer than the control from June 2013 to April 2014 (*P* < 0.01).

Melatonin implants stimulated cashmere growth for which regressions were calculated (Table 2). Initiation date of the cashmere growth cycle for treated goats was earlier than control goats (May 18, 2013 vs. July 2, 2013; *P* < 0.01), while the cessation date for growth was similar to that of the control goats (March 22, 2014 vs. March 27, 2014). The duration of cashmere growth (307 vs. 270 days) was longer in melatonin-treated goats than in control goats (*P* < 0.01). No significant differences were observed in cashmere growth rate between treated and control goats.

**Table 2:** Effect of melatonin implantation on the duration of cashmere growth and cashmere growth rate in Inner Mongolian cashmere goats^1^

Group	Initiation date	Cessation date	Duration of growth, days	Cashmere growth rate, mm/d
Control	July 2, 2013^a^	March 27, 2014	270^b^	0.36
Melatonin	May 18, 2013^b^	March 22, 2014	307^a^	0.37
S.E.M^2^	4.03	6.03	5.14	0.015
*P*-value	<0.01	0.42	<0.01	0.29

^1^Values are means of nine replicates per treatment. Means with different superscripts (a and b) within a column differ (*P* < 0.05)

^2^S.E.M = Standard error of the mean

### Reproductive performance

The effect of melatonin treatment on reproductive performance is presented in Table 3. The kidding date, litter size, and birth weight were not significantly affected by treatment.

**Table 3:** Effect of melatonin implantation on the reproductive performance of Inner Mongolian cashmere goats^1^

Item	Treatment		S.E.M^3^	*P*-value
	Control	Melatonin		
Kidding date	March 14, 2014	March 16, 2014	5.10	0.68
Litter size^2^	1.13	1.00	0.13	0.35
Birth weight, kg	3.22	3.14	0.29	0.77

^1^Values are means of nine replicates per treatment

^2^Litter size = mean no. of kids per birth

^3^S.E.M = Standard error of the mean

## Discussion

Our study showed that the final BW and average daily gain were not affected by melatonin implantation which is in accordance with the findings of Liu et al. [13] and Yue et al. [18]. Our study showed that use of melatonin implants initiated cashmere growth earlier than normal and increased cashmere yield by 34.5 % and maximum length by 21.3 % during the following year. Moreover, there was no early shedding observed in the treatment group.

Research on the effects of melatonin implantation on productive parameters in cashmere goats have mainly focused on cashmere production. Previous studies have shown that exogenous melatonin has a positive role on cashmere production [9–15, 19–22]. However, none of the previous studies demonstrated an improvement in cashmere yield of a similar magnitude to that found in the current study.

One factor that could account for the discrepancy in the magnitude of the response to melatonin is the timing of implantation. For example, Liu et al. [13] demonstrated that melatonin implantation (1.2 mg/kg BW) in late May (cashmere non-growing period) initiated cashmere growth earlier than normal but increased cashmere yield during the following year by only 7.13 %. Their subsequent experiment on melatonin implantation, administrated with two doses in late April in Inner Mongolian cashmere goats, Liaoning cashmere goats and a cross between the two, confirmed the aforementioned result of cashmere growth ahead of schedule in melatonin-treated goats. However, the cashmere in all of the melatonin-treated goats was shed during the period from late August to early September and another cashmere growth cycle followed but the cashmere yield during the following year was not affected by melatonin implantation [14].

Other studies may have implanted too late. Yue et al. [15] found that melatonin implants (2 mg/kg BW; implant insertion every 2 months) from late June to late November increased cashmere yield by only 7.8 %. Chang [21] reported that continuous melatonin treatment (2 mg/kg BW; implant insertion every 2 months) throughout the year since late July improved the cashmere yield by only 7.4 % in Inner Mongolian cashmere goats. No early shedding were observed in all of the melatonin-treated goats during the period from late August to early September in the researches of Yue et al. [15] and Chang [21]. In the current study, melatonin (2 mg/kg BW) was implanted again in June after the first implantation in April, which was probably the main reason resulting in continued cashmere growth without early shedding.

Cashmere fibre diameter is the most important factor to define the value per unit weight of cashmere fleece. Therefore, increasing cashmere production and maintaining low cashmere fibre diameter are typically associated with higher economic returns. The effects of melatonin implants on cashmere fibre diameter have not been consistent. Our study showed that melatonin implants decreased cashmere fibre diameter, which is in agreement with the finding of Wang et al. [23]. In contrast, Wuliji et al. [20] reported that melatonin treatment increased the fibre diameter in Spanish goats while other studies have shown that administration of melatonin has no effects on cashmere fibre diameter in Inner Mongolian cashmere goats [13, 15, 21], Liaoning cashmere goats [22] or New Zealand cashmere goats [19]. Differences in the response to melatonin implantation on cashmere fibre diameter in the above mentioned reports may be related to differences in goat breed, the applied dose of melatonin and the specific time of melatonin treatment.

The increased cashmere yield observed in the present study can be attributed to the extension of the cashmere growth cycle resulting in longer fibre length. The extension of the cashmere growth cycle in melatonin-treated goats was mainly due to cashmere growth from May to August, because there were no differences in the growth rate and cessation date between the control and treated goats. The calculated initiation date of cashmere growth for the control goats was on July 2, 2013 and ceased on March 27, 2014 with a total cashmere growth cycle of 270 days. Melatonin implants extended the cashmere growth phase, causing the initiation of cashmere growth to occur 45 days earlier, with an average initiation date of May 18, 2013 and cessation date of March 22, 2014. The cashmere growth cycle was 307 days.

The finding of an extension in the cashmere growth cycle agrees with previous research. From experiments conducted during two consecutive years, Welch et al. [12] concluded that melatonin implanted during the cashmere non-growing period could induce cashmere growth and extend the cashmere growth phase. Klören and Norton [9] also found that melatonin supplementation during the cashmere non-growing period extended the cashmere growth phase as did Liu et al. [13] and Liu et al. [14]. Kennaway et al. [24] suggested that constant melatonin availability during periods of long day length may have consequences similar to a short day length in cashmere goats.

Reproductive activity in goats shows an annual pattern with a period of breeding activity occurring when day length is decreasing, and the onset of seasonal anoestrus when day length is increasing [25]. Investigation of the effects of exogenous melatonin on the reproductive performance has seldom been conducted in cashmere goats. Chang [21] reported that continuous melatonin treatment did not exert any significant effect on reproductive performance in Inner Mongolian cashmere goat. Our results show similar findings in that reproductive performance was not affected by melatonin implantation from late April to late August (anestrous season), which indicates that melatonin implants during the anestrous season did not affect the reproductive performance of cashmere goats during the following year.

## Conclusions

We conclude that melatonin supplementation increased cashmere yield by extending the cashmere growth cycle and increasing maximum fibre length. Melatonin implants decrease the cashmere fibre diameter without changing growth rate or reproductive performance. Thus, melatonin implants (2 mg/kg BW) on two occasions (in late April and June) is a practical and effective method for improving cashmere production in seasonal-type cashmere goats.

## References

[1] McCarthy BJ (1998). Specialty animal fibres. Textiles.

[2] Tang MT, Zhang WP, Zhou H, Fei J, Yang J, Lu WM (2014). A real-time PCR method for quantifying mixed cashmere and wool based on hair mitochondrial DNA. Text Res J.

[3] Shamsaddini-Bafti M, Salehi M, Maghsoudi A, Tehrani AM, Mirzaei F, Momen SMS (2012). Effect of sex and rearing system on the quality and mineral content of fiber from raeini cashmere goats. J Anim Sci Biotechnol.

[4] Seki Y, Yokohama M, Wada K, Fujita M, Kotani M, Nagura Y (2011). Expression analysis of the type I keratin protein keratin 33A in goat coat hair. Anim Sci J.

[5] Zhou HM, Allain D, Li JQ, Zhang WG, Yu XC (2003). Effects of non-genetic factors on production traits of Inner Mongolia cashmere goats in China. Small Rumin Res.

[6] Liu HY, Li N, Jia CL, Zhu XP, Jia ZH (2007). Effect of the polymorphisms of keratin associated protein 8.2 gene on fibre traits in Inner Mongolia cashmere goats. Asian Austral J Anim.

[7] Zhang W (2011). Study on the fur growth rules of Inner Mongolia cashmere goats.

[8] Teh TH, Jia ZH, Ogden KD, Newton GR (1991). The effects of photoperiod and melatonin implant on cashmere production. J Anim Sci.

[9] Klören WRL, Norton BW (1995). Melatonin and fleece growth in Australian cashmere goats. Small Rumin Res.

[10] Betteridge K, Welch RAS, Pomroy WE, Lapwood KP, Devantier BP (1987). Out of season cashmere growth in feral goats. Proceedings of the second international cashmere conference.

[11] Litherland AJ, Paterson DJ, Parry AL, Dick HB, Staples LD (1990). Melatonin for cashmere production. Proc N Z Soc Anim Prod.

[12] Welch RAS, Gurnsey MP, Betteridge K, Mitchell RJ (1990). Goat fibre response to melatonin given in spring in two consecutive years. Proc N Z Soc Anim Prod.

[13] Liu JC, Gui R, Zhao QS (1994). Effects of melatonin on the cashmere growth and production in Inner Mongolian cashmere goats during the cashmere non-growing period. Chin J Zool.

[14] Liu JC, Yin XZ, Fang TQ (1998). Effect of melatonin on the cashmere growth and production in cashmere goats. Chin J Zool.

[15] Yue CW, Zhang W, Kong XH, Liu HY, Jia ZH (2007). Effect of melatonin on cashmere performance in Inner Mongolia white cashmere goats. Chin J Anim Sci.

[16] Norton BW, Klören WRL (1995). Measurement of the components of the cashmere growth cycle in Australian cashmere goats. Small Rumin Res.

[17] SAS Institute (1999). SAS OnlineDoc®, version 8.

[18] Yue CW, Du LX, Zhu XP, Kong XH, Zhang W, Jia ZH (2007). Skin deiodinase profiles after melatonin manipulation in Chinese Inner Mongolia Cashmere Goats. Asian Austral J Anim.

[19] O’Neill K, Litherland AJ, Paterson DJ (1992). Melatonin for cashmere production in breeding does. Proc N Z Soc Anim Prod.

[20] Wuliji T, Litherland A, Goetsch AL, Sahlu T, Puchala R, Dawson LJ (2003). Evaluation of melatonin and bromocryptine administration in Spanish goats: II. Effect on seasonal cashmere growth, yield and fiber characteristics of does. Small Rumin Res.

[21] Chang ZL (2010). Study on effect of constant-release melatonin implants on the cashmere growth traits of cashmere goats and related gene.

[22] Cong YY, Deng HW, Feng YL, Chen Q, Sun Y (2011). Melatonin implantation from winter solstice could extend the cashmere growth phase effectively. Small Rumin Res.

[23] Wang LF, Lu DX, Sun HZ, Zhao XY, Shan D (2006). Effects of photoperiod and melatonin on nitrogen partitioning and cashmere growth in Inner Mongolia White Cashmere goats. Sci Agric Sin.

[24] Kennaway DJ, Gilmore TA, Seamark RF (1982). Effects of melatonin implants on the circadian rhythm of plasma melatonin and prolactin in sheep. Endocrinology.

[25] Zarazaga LA, Celi I, Guzmán JL, Malpaux B (2010). Melatonin concentrations in the two jugular veins, and relationship with the seasonal reproductive activity in goats. Theriogenology.

